# Pharmacological characterisation of the effort for reward task as a measure of motivation for reward in male mice

**DOI:** 10.1007/s00213-023-06420-9

**Published:** 2023-07-21

**Authors:** Caterina Marangoni, Melissa Tam, Emma S. J. Robinson, Megan G. Jackson

**Affiliations:** School of Physiology, Pharmacology & Neuroscience, Biomedical Sciences Building, University Walk, Bristol, BS8 1TD UK

**Keywords:** Effort-based decision-making, Mice, Motivation, Model, Research Domain Criteria

## Abstract

**Rationale:**

Motivational deficits are a common symptom shared across multiple psychiatric and neurodegenerative disorders. Effort-based decision-making tasks are a translatable method for assessing motivational state. Much of the preclinical validation of the task derives from acute pharmacological manipulations in rats. However, mice currently offer a greater genetic toolkit to study risk genes and phenotypic models. Despite this, there is limited characterisation of their behaviour in this type of motivation task.

**Objectives:**

Here, we investigate the effort for reward (EfR) task as a measure of motivational state in mice using drugs previously shown to modulate effort-based decision-making in rats and humans.

**Method:**

Using male C57bl/6j mice, we test the effects of drugs which modulate DA transmission. We also test the effects of CP101-606 which does not act directly via DA modulation but has been shown to exert beneficial effects on motivational state. Finally, we test the sensitivity of the task to a chronic corticosterone (CORT) treatment.

**Results:**

Amphetamine, methylphenidate, and CP101606 in mice increased high-effort responses for high-value reward, while administration of haloperidol decreased high-effort responses. Surprisingly, tetrabenazine had no effect at the doses tested. Chronic, low-dose CORT consumption did not alter task performance.

**Conclusion:**

These data suggest that the EfR task is sensitive to acute dopaminergic modulation and NR2B selective antagonism in mice. However, it may lack sensitivity to non-acute phenotypic models. Further work is required to demonstrate the utility of the task in this context.

**Supplementary Information:**

The online version contains supplementary material available at 10.1007/s00213-023-06420-9.

## Introduction

It has become increasingly recognised that deficits in motivation for reward are a common symptom of numerous neuropsychiatric and neurological conditions, including major depressive disorder (MDD), apathy syndrome, and schizophrenia (Fervaha et al. 2013; Marin 1991; Yang et al. 2014). Observed homology in symptoms across multiple conditions has encouraged a growing movement towards addressing complex disorders in a symptom-based approach to the benefit of translational drug development. The Research Domain Criteria (RDoC) supports this approach, by emphasising the underlying neurobiological mechanisms of specific symptom sets, which may be shared across multiple disorders (Cuthbert 2015; Woody and Gibb 2015; Nusslock and Alloy 2017). The relevance of motivational deficits has been recognised within this new matrix and has been included as part of the positive valanced system.

Effort-based decision-making tasks (EBDM), also referred to as concurrent choice tasks, are a translatable method for assessing motivational state. In these paradigms, the subject is concurrently presented with a high-effort-high-value reward option or a low-effort-low-value reward option. In most versions of the task, the individual can freely choose between these two options throughout the session. For preclinical studies, EBDM tasks exist in various formats and contexts, including a T maze barrier task, where effort is required to scale a vertical barrier and an operant lever press/nose poke task. In the operant effort for reward (EfR) task, the rodent can choose to make an effortful number of responses to receive a high-value reward (e.g. sucrose enriched food pellet) or consume freely available lab chow. Increased willingness to exert effort for a higher value reward is indicative of a higher motivational state. Both versions of the task have been investigated in rats, with studies showing sensitivity to systemic and targeted manipulations of the mesolimbic dopaminergic system (Salamone et al. 1994; Nunes et al. 2013; Cousins et al. 1996). Reduction in dopaminergic (DA) transmission using D_2_ receptor antagonist haloperidol and vesicular monoamine transporter 2 (VMAT-2) inhibitor tetrabenazine (TBZ) reduces high-effort choices, while potentiation of DA transmission using psychostimulants such as amphetamine increases them (Floresco et al. 2008). There is also evidence to suggest that the task is sensitive to manipulations of other neurochemical systems such as gamma-aminobutyric acid (GABA) and acetylcholine and involve multiple regions of the mesolimbic network including the prefrontal cortex and basolateral amygdala (Salamone et al. 2015).

Unusually for the field, the paradigm has been back translated for use in patient populations. The Effort Expenditure for Reward task (EEfRT) is a multi-trial task associated with varying levels of monetary reward. Greater amounts of money are associated with higher effort (more button presses with less dominant finger), and lower amounts of money are associated with lower effort (less button presses with dominant finger). This task has been shown to be sensitive to motivational deficits in multiple clinical populations, including patients with MDD and schizophrenia, where behaviour is shifted towards the lower effort, lower monetary value option (Treadway et al. 2012; McCarthy et al. 2016). In addition, it has been shown to be sensitive to acute DA modulation, where administration of amphetamine and methylphenidate increased high-effort responding in attention-deficit/hyperactivity disorder (ADHD) patient populations (Wardle et al. 2011; Addicott et al. 2019).

In this study, we investigated the EfR task as a translational measure of motivational state in mice. Currently, there is a greater toolkit available for mice with which to create neurodegenerative and neuropsychiatric models based on established risk genes. A future focus on using models of disease states where motivational deficit is a core symptom rather than an acute pharmacological approach may engage more relevant mechanisms and thus have greater translational validity. However, a significant proportion of the preclinical task validation has been completed in rats, and it is unclear whether the same behavioural findings can be extended to mice. Mice and rats differ considerably at the behavioural level, including different social behaviours, reward-related behaviours (Lore and Flannelly 1977; Schmid-Holmes et al. 2001; Kummer et al. 2014), and task-related stress (Ellenbroek and Youn 2016). We first investigate the effects of acute administration of drugs which modulate DA transmission and have previously been shown to modulate EfR behaviour in rats. We test the effects of CP101-606 which does not act directly via DA modulation but has been shown to exert beneficial effects on motivational state (Gilmour et al. 2009). Finally, we test the sensitivity of the task to a chronic corticosterone (CORT) treatment. While there have been relatively few studies of EBDM in phenotypic models, motivational deficit in healthy ageing (Jackson et al. 2021) as well as a catechol-o-methyltransferase (COMT) model of schizophrenia (Yang et al. 2020a) in mice has been reported.

## Methods

### Subjects

Sixteen male C57bl/6J mice were purchased from Envigo UK. At the start of experimentation, they were aged 7 weeks and weighed 27.4–31.6 g, and by the end of experimentation, they were aged 54 weeks and weighed 30.5–40.3 g. They were singly housed to minimise any impacts of aggression and associated psychosocial stress in enriched open-top Techniplast 1284 conventional caging, with a small red house, cardboard tube, cardboard tube suspended from the ceiling, a wooden chew, nestlet, and bedding material. Standard laboratory chow (Purina, UK) and water were provided ad libitum apart from during training and testing, where mice were mildly food restricted. Food was removed 5 h before testing and replaced 1 h after testing. Mice were housed in temperature-controlled conditions (~ 21 °C) and a 12:12 reverse lit light cycle, with lights OFF at 9.30 am, and lights ON at 9.30 pm. Mice underwent behavioural testing in their active phase between 1 and 4 pm (ZT15.5–18.5). Weights were monitored at least once a week and maintained to at least 85% of their free-feeding weight relative to their standard growth curve. Mice did not start testing until 1 week after arrival and were habituated to cup handling before training began. To complete the chronic corticosterone study, a second cohort of 16 mice of the same strain and sex was housed, handled, habituated, and tested under the same conditions. At the start of experimentation, they were aged 7 weeks and weighed 23–27 g. By the end of experimentation, they were aged 33 weeks and weighed 30.5–34.1 g. Sample sizes were based on detecting a large effect size (~ 1.0) with mean difference and variance based on previous behavioural studies using a similar task and acute/chronic pharmacological manipulations (Griesius et al. 2020; Jackson et al. 2021). All experiments were performed in accordance with Animals Scientific Procedures Act (ASPA, UK) 1986 and were approved by the University of Bristol Animal Welfare and Ethical Review Body (AWERB).

### Operant training

Operant training was consistent with the protocol reported in (Jackson et al. 2021). Mice were trained in sound-proofed operant boxes (MedAssociates) which were run on Klimbic software. The inside of the box was lit using dim red LED lights so that mouse behaviour could be videoed in perceived darkness. The operant box consisted of two nose poke apertures where operant responses could be made and a centrally located food magazine. The food magazine was connected to a reward pellet dispenser (20 mg, rodent tablet, TestDiet, Sandown). An Lifecam HD 3000 webcam was positioned on the ceiling of the operant box, with the whole interior of the box within view. The webcams were controlled using an in-house developed Python script, which allowed for multiple webcams to record in parallel, and be saved in distinct files. Mice were only videoed during acute pharmacology test sessions (further detail below).

Training was completed in three stages. In all stages of training, mice underwent one 30-min session per day. In the first stage, mice learned to associate the magazine with the delivery of reward. Every 40 s, a reward pellet was automatically dispensed into the magazine. This lasted for 2 days. In the second stage of training, mice underwent a continuous reinforcement (CRF) schedule of training. Here, a response could be made in either of the nose poke apertures, resulting in the delivery of a reward pellet into the magazine. When the reward was delivered, the magazine was illuminated until the pellet was collected. This training stage lasted 5 days. In the third stage of training, mice advanced through ascending fixed ratios of reinforcement (FR). Here, only one nose poke aperture was active, either the left or right, the position of which counterbalanced across the cohort. Mice progressed through FR1, 2, 4, and 8, where the number refers to the number of nose pokes required for the delivery of a single reward pellet. For example, FR8 refers to 8 nose pokes for one reward pellet. Mice advanced through each FR stage when they completed two consecutive days of 30+ trials, with the exception of FR8, where 20+ trials were required. FR8 was selected as the final stage as it has previously been shown to be a sufficiently high enough level of effort to detect motivational deficit (Jackson et al. 2021). A trial refers to the delivery of reward following the required number of nose pokes necessary. Mice were moved through the FR stages on an individual basis. Once all mice had completed FR training, testing began (training data available in supplementary results (S1). For the final acute pharmacological studies, the FR required was dropped to 4, to account for potential age-induced reduction in responses.

### Effort for reward task

**Fig. 1:**
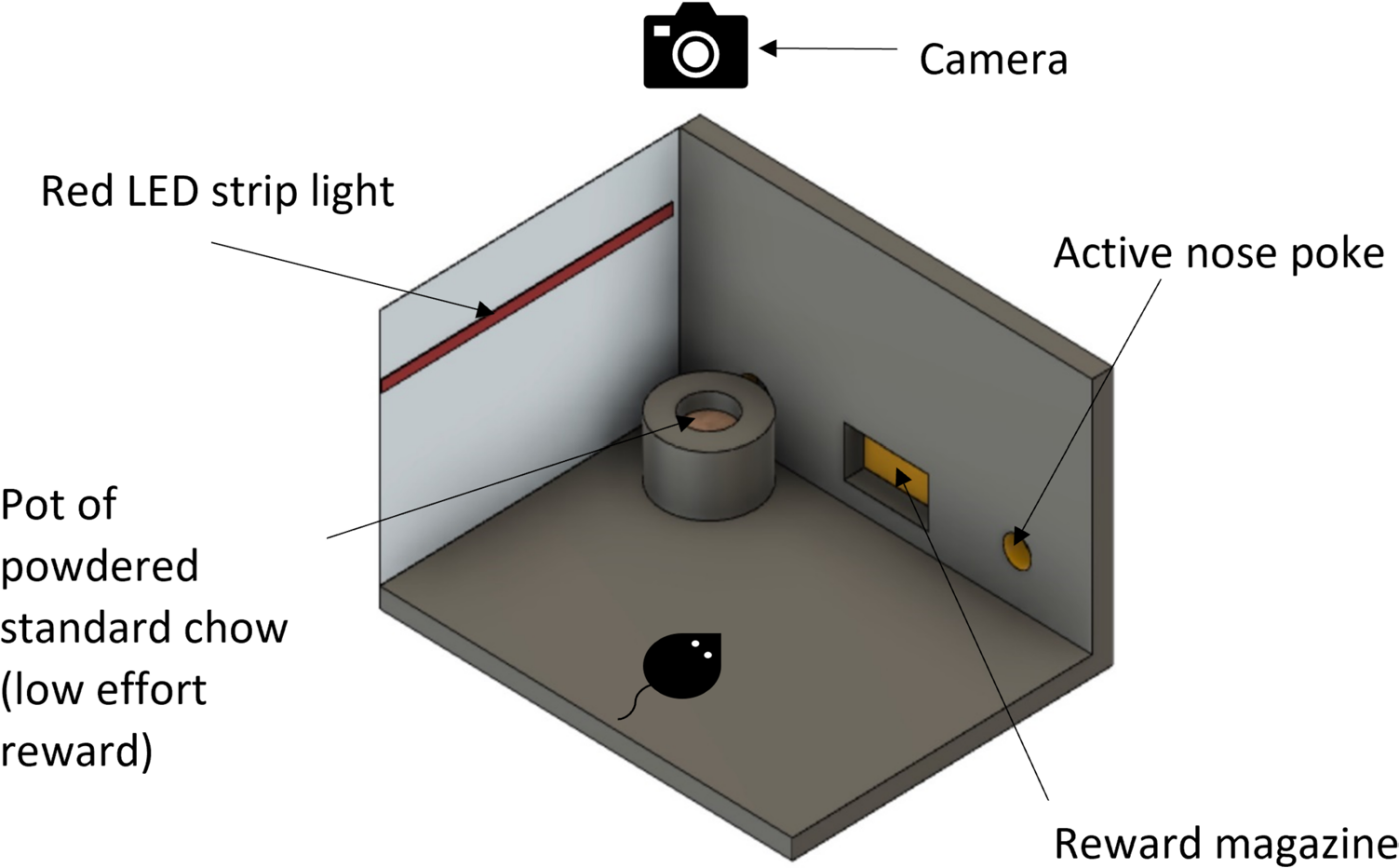
Effort for reward task set up. The mouse is given the option to complete a fixed number of nose pokes for a reward pellet (high-effort, high-reward) or consume freely available lab chow accessed from a bowl placed in the corner. The operant box was illuminated by a low-level red LED strip, and a webcam was suspended above the box from the ceiling

The effort for reward task consists of an FR8 session (or FR4 in later sessions, described below), with the addition of a bowl of standard laboratory chow in powdered form positioned in front of the inactive nose poke aperture (Fig. 1). The chow could be accessed by a 0.5-inch hole in the lid. This was to allow easy reach of the chow but prevent excess digging behaviour. The requirement of 8 nose pokes for the delivery of a reward pellet represents the high-effort, high-value reward option. The freely available lab chow represents the low-effort, low-value reward option. Throughout the session, the mouse could freely choose whether to exert higher effort for higher value reward by responding at the nose poke or consume the low-effort, low-value reward from the bowl. The number of trials completed was recorded, as well as the amount of chow consumed, by measuring the weight of the chow bowl before and after the session in grams. We also investigated if video analysis of chow consumption-related behaviours could provide additional information about effort-related choice behaviour. Behaviour at the chow bowl was videoed and scored using a novel, in-house developed application. We are making this application freely available for use https://github.com/dandovi/efr-video-analysis-tool. Detailed definitions of the outputs recorded are described in (Table 1). Before pharmacological testing, mice underwent a test session to habituate them to the presence of chow bowl in the operant box.

**Table 1:** Output measures of the effort for reward task. Italicised entries refer to output measures relating to chow bowl from video analysis

Measure	Description
Number of trials	The number of completed high-effort trials which resulted in high-reward delivery.
Chow consumed	The amount of chow eaten from the bowl within the session (grams).
*Chow bouts*	*The number of times the mouse visits the bowl throughout the session.*
*Latency to chow bowl*	*The time taken for the mouse to interact with the chow bowl for the first time within the session (s).*
*Duration at chow bowl*	*The average duration of the bouts at the chow bowl (s).*
Latency to complete first trial	Time taken for the mouse to complete the first trial to receive a reward pellet (s).

### Experimental design

All experiments were performed with the experimenter blind to treatment. In the case of acute pharmacological experiments, each mouse received each dose and appropriate vehicle control in a fully randomised, within-subject design over four test sessions with a final *n* of 16 mice for each dose. For the chronic corticosterone experiment, half the mice received normal water, while the other half received treated water (more details below). A second cohort of 16 mice were trained and split into control and treated groups. Thus, there were 16 mice for control and treated groups in a between-subject design. Following the end of the chronic corticosterone study, the corticosterone-treated mice were killed, and the mice from the two cohorts were combined to complete the remaining acute studies (see supplementary figure 1 (S2) for timeline). It should be acknowledged that in an effort to reduce number of animals used across the study, advancing age of the mice and repeated use of the task may impact on task performance. This was mitigated where possible by inclusion of a vehicle control group and avoiding clustering the use of drugs relating to a particular system or expected effect at the start or end of the study.

Mice were administered drugs either intraperitoneally (I.P.), sub-cutaneously (S.C.) or orally with a dose volume of 10 ml/kg and handled for dosing using refined methods (Davies et al. 2022). Pretreatment times (*t*-) and doses were based on previously published experiments using the effort for reward task and as much as possible, to align with clinically relevant doses (Griesius et al. 2020; Stuart et al. 2017; Floresco et al. 2008; Gilmour et al. 2009). Drugs for acute pharmacology were dissolved in either 0.9% sterile saline (amphetamine, methylphenidate) or 1% dimethyl sulfoxide (DMSO), 2% cremophore, 97% sterile saline (haloperidol, CP101606, and tetrabenazine).

### Acute pharmacology

Baseline FR8 sessions were conducted on a Mon and Thurs, while test sessions were conducted on a Tues and Fri. This was to ensure parity of behavioural experience across the week, and allow for sufficient time to allow for drug washout between test sessions (see supplementary figure 2A (S3) for design). Each acute pharmacological study lasted 2 weeks (4 test sessions). The following drugs were tested: haloperidol (0.01, 0.03, and 0.1 mg/kg, s.c., *t*—60 min, Sigma UK), amphetamine (0.1, 0.3, and 1 mg/kg, i.p, *t*—15 min, Sigma UK), CP101606 (1, 3, and 10 mg/kg, s.c, *t*—30 min, Axon Medchem), methylphenidate (1, 3, and 10 mg/kg, oral, *t*—15 min, Sigma UK), and tetrabenazine (0.1, 0.3, and 1 mg/kg, s.c., *t*—90 min, Sigma UK) (Table 2).

**Table 2:** Summary table of drugs used in the task. *DA*, dopaminergic; *DAT*, dopamine active transporter; *NMDAR*, N-methyl-D-aspartate receptor; *VMAT-2*, vesicular monoamine transporter 2

Drug	Doses used (mg/kg)	Pretreatment time (mins)	Primary mechanism of action at doses selected
Haloperidol	0.01, 0.03, and 0.1	60	Antagonism of D2 receptors resulting in reduced DA transmission.
Amphetamine	0.1, 0.3, and 1.0	15	Stimulation of DA release and inhibition of DA uptake via VMAT-2 interaction resulting in increased DA transmission.
CP101-606	1.0, 3.0, and 10.0	30	Modulation of glutamate signaling at GluN2B containing NMDARs.
Methylphenidate	1.0, 3.0, and 10.0	15	Inhibition of DAT, reducing DA uptake and resulting in increased DA transmission.
Tetrabenazine	0.1, 0.3, and 1.0	90	Inhibition of monoamine uptake into vesicles of presynaptic neurons by binding VMAT-2, resulting in decreased DA transmission.

### Chronic corticosterone treatment

Half of the mice were given water treated with corticosterone (HBC complex, Sigma) (2.5 mg in 100 ml). Mice on average drank ~ 5 ml water/day and weighed on average 35 g; thus, this is equivalent to ~ 3.5 mg/kg per day. The other half were given normal drinking water. Water was provided in dark bottles to protect CORT-treated water from the light. Water was replaced every 2 days and weighed daily. Mice were given treated/normal water for 14 days. They then underwent 4 baseline sessions of FR8 and a final EfR test session. They were maintained on CORT treatment throughout this third week. Thus, mice underwent 18 days of treatment before the testing session (supplementary figure 2B (S2)).

### Statistical analysis

*Acute pharmacology studies*: data were tested for normality using a Shapiro-Wilk test. Where data were normally distributed, a repeated measure one-way ANOVA (Geisser-Greenhouse corrected) was used for all output measures. Where a significant main effect of drug was observed (*p* < 0.05), Dunnett’s post hoc comparisons were carried out. Where data violated normality, the Friedman test was applied. Where significant main effects were observed, Dunn’s post hoc analysis was conducted. Trend level main effects (*p* < 0.1) were reported but not further analysed. Outliers were defined as values 2 standard deviations away from the mean. For each output measure, outliers were replaced with the group mean to permit repeated measure analysis. Where significant effects are observed, replaced outliers are reported in the results. In the cases where latency to approach chow bowl or complete first was 0 due to lack of engagement, the full trial time (1800 s) was used. For the final tetrabenazine and methylphenidate studies, *n* = 14 mice were used due to 2 showing signs of stereotypic behaviour.

*Chronic corticosterone*: data were tested for normality using a Shapiro-Wilk test. Where data was normally distributed, an unpaired *t*-test was performed on all output measures. Where data violated normality, a Mann-Whitney *U* test was performed. Data 2SDs away from the mean were excluded. This is reported in the results.

Analysis and graph construction were performed using GraphPad Prism v 9.4.0.

## Results

### Haloperidol but not tetrabenazine reduces high-effort selection in the effort for reward task

**Fig. 2:**
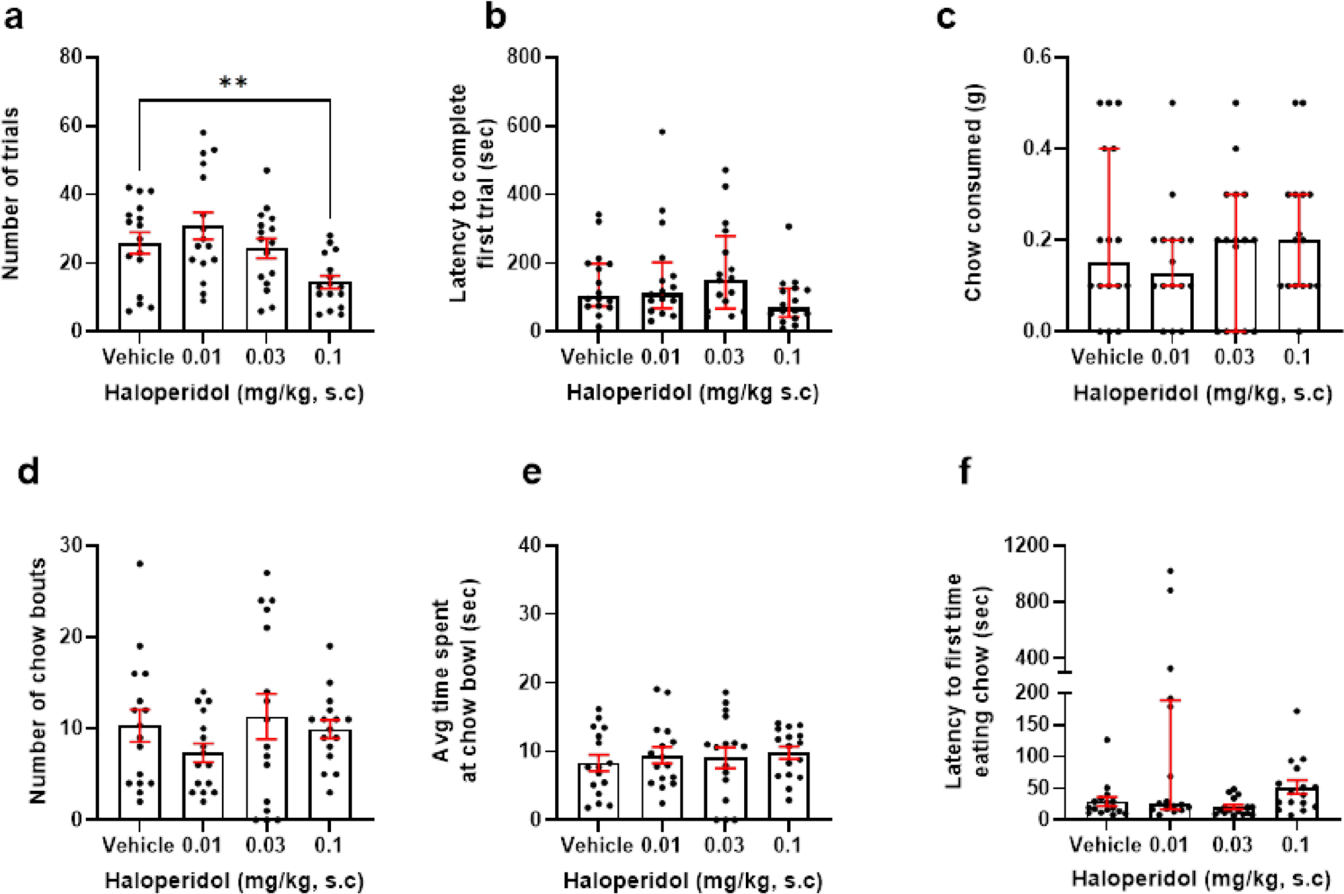
Haloperidol reduces high-effort option selection in a dose-dependent manner. Mice were dosed with 0.01–0.1 mg/kg haloperidol and underwent the EfR task. **a** 0.1 mg/kg haloperidol reduced number of high-effort, high-value trials performed (*p* < 0.01). **b** Haloperidol did not affect latency to complete first trial, **c** chow consumed, **d** number of chow bouts or **e** average time spent at the chow bowl (*p* > 0.05), or **f** latency to first time eating chow (*p* > 0.05). **p < 0.01. Bars are mean ± SEM or median ± interquartile range (**b**, **c** + **f**)

The effect of 0.01–0.1 mg/kg haloperidol on effort for reward task performance was analysed. There was a main effect of drug on number of trials completed (*F*_(2.292, 34.38)_ = 9.896, *p* = 0.0002, RM one-way ANOVA). Post hoc analysis revealed that mice treated with 0.1 mg/kg haloperidol completed less trials versus vehicle (*p* = 0.0023) (Fig. 2a). There was no effect of haloperidol on latency to complete first trial, amount of chow consumed, average time spent at the chow bowl, or number of bouts at the chow bowl (*p* > 0.05) (Fig. 2b–e). However, there was a main effect of drug on latency to approach the chow bowl (*X*_2_ = 14.63, *p* = 0.0022). Post hoc analysis revealed that mice treated with 0.01 mg/kg trended towards a greater latency to approach the chow bowl for the first time versus vehicle (*p* = 0.0598) (Fig. 2f). Here, *n* = 1 outliers were replaced from the vehicle and 0.01 mg/kg groups, and *n* = 3 and 2, respectively, for the 0.03 and 1 mg/kg groups.

**Fig. 3:**
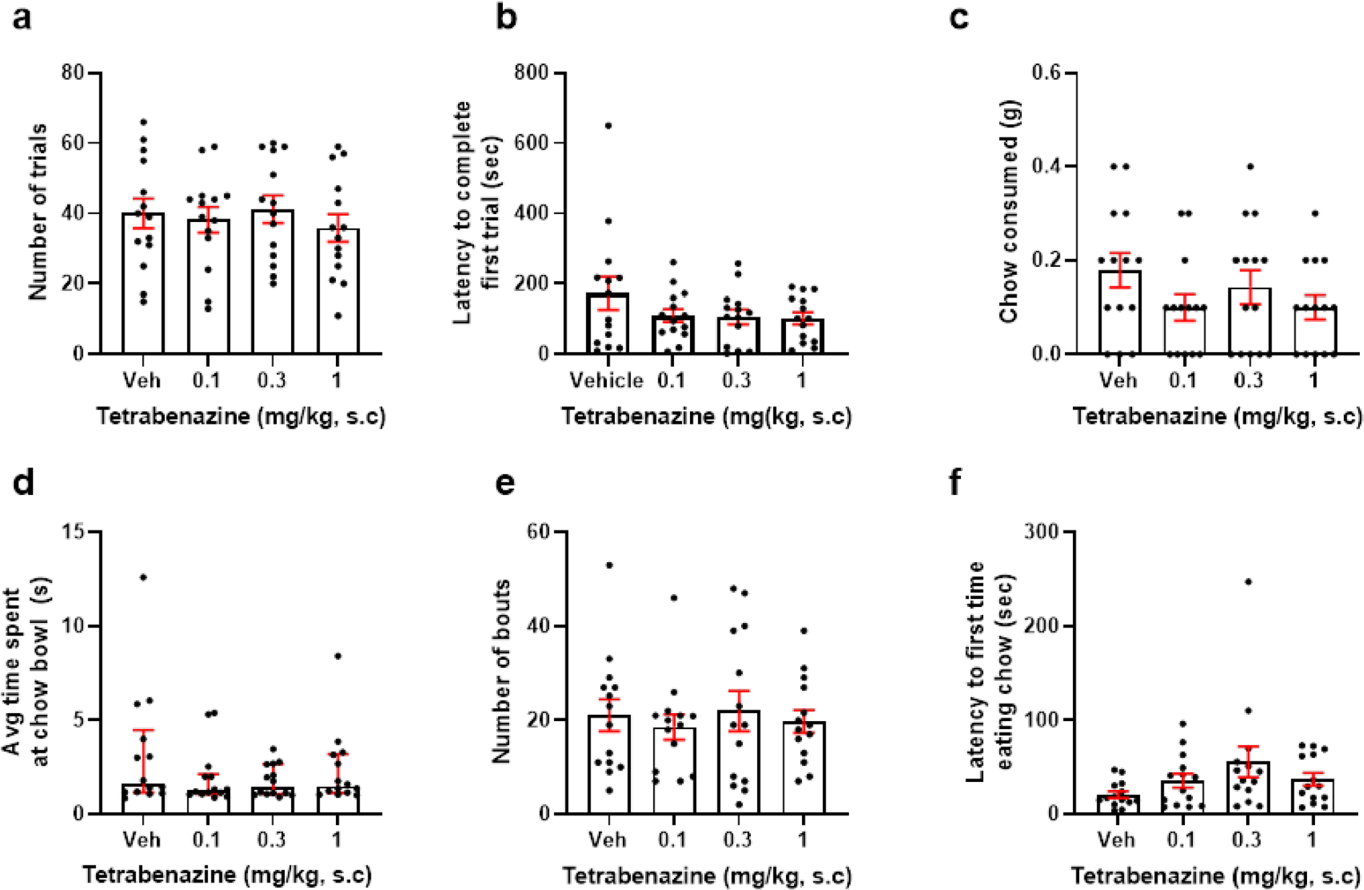
Tetrabenazine has no effect on EfR task performance. Mice were dosed with 0.1–1 mg/kg tetrabenazine and underwent the EfR task. **a** Tetrabenazine had no effect on number of trials, **b** latency to complete first trial, **c** chow consumed, **d** number of chow bouts or **e** average time spent at the chow bowl, or **f** latency to first time eating chow (*p* > 0.05). Bars are mean ± SEM or median ± interquartile range (**d**)

There was no effect of 0.1–1 mg/kg tetrabenazine in any of the effort for reward task parameters (*p* > 0.05) (Fig. 3a–f).

### Amphetamine, CP101606, and methylphenidate increase high-effort selection in the effort for reward task

**Fig. 4:**
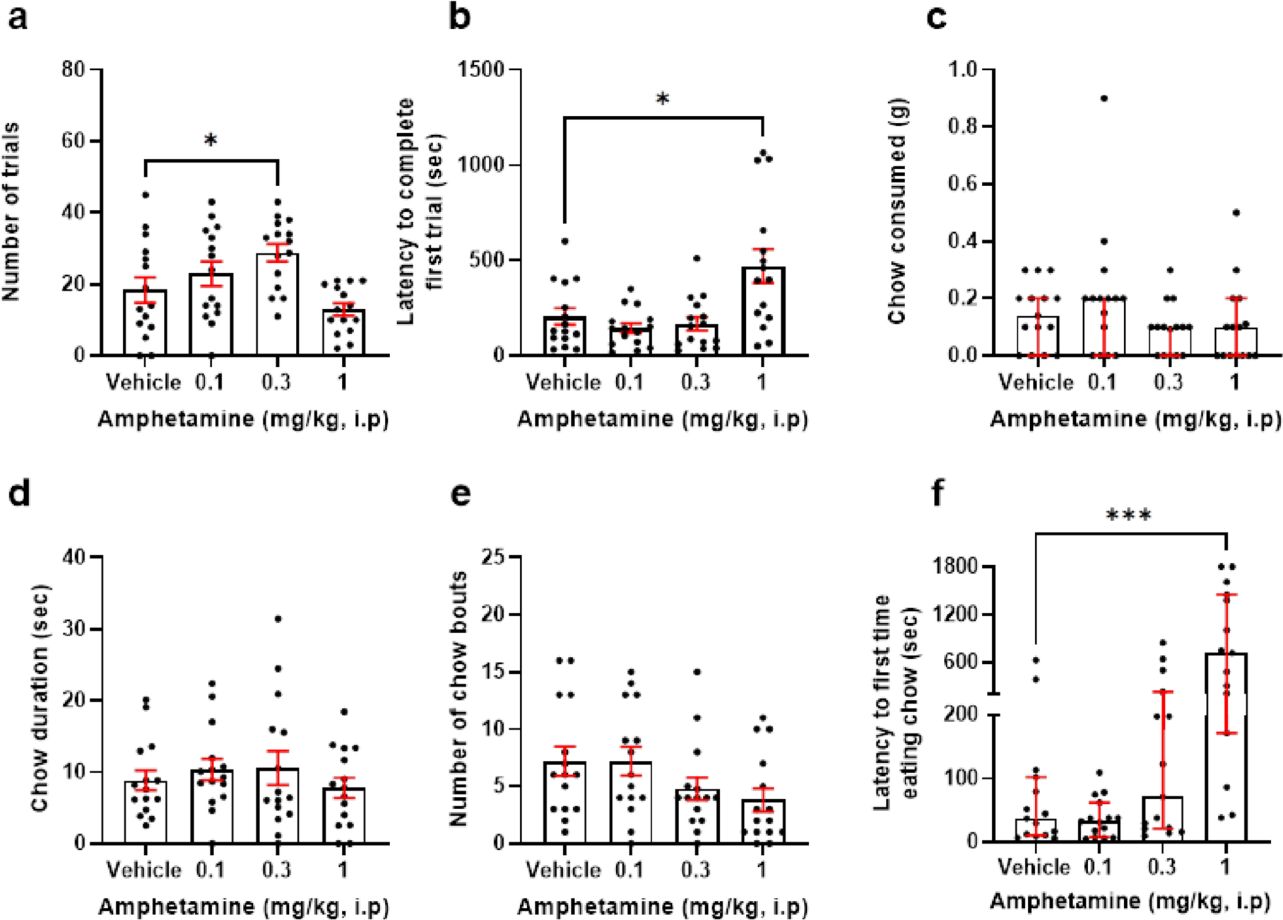
Amphetamine increases high-effort option selection in a dose-dependent manner. Mice were dosed with 0.1–1 mg/kg amphetamine and underwent the EfR task. **a** 0.3 mg/kg amphetamine increased number of high-effort, high-value trials performed (*p* < 0.05). **b** 1 mg/kg amphetamine increased latency to complete first trial. **c** It had no effect on chow consumed, **d** number of chow bouts, or **e** average time spent at the chow bowl (*p* > 0.05). **f** 1 mg/kg increased latency to first time eating chow (*p* < 0.05). *p < 0.05, ***p < 0.001. Bars are mean ± SEM or median ± interquartile range (**c**, **f**)

The effect of 0.1–1 mg/kg amphetamine on effort for reward performance was analysed. There was a main effect of drug on number of trials completed (*F*_(2.114, 29.6)_ = 6.914, *p* = 0.003, RM one-way ANOVA). Post hoc analysis revealed that 0.3 mg/kg increased number of trials relative to vehicle (*p* = 0.0235) (Fig. 4a). *N* = 1 outliers were replaced from the 0.3 and 1 mg/kg groups. There was also a main effect of amphetamine on latency to complete first trial (*F*(1.877, 28.16) = 10.76, *p* = 0.0009, RM one-way ANOVA). Post hoc analysis revealed that 1 mg/kg increased latency to complete first trial (*p* = 0.0158) (Fig. 4b). Here, *n* = 1 outliers were replaced across all doses. There was no effect of amphetamine treatment on amount of chow consumed and time spent at the chow bowl (*p* > 0.05) (Fig. 4c, d). However, there was a trend towards amphetamine reducing number of bouts at the chow bowl (*F*_(2.6, 36.4)_ = 2.764, *p* = 0.063, RM one-way ANOVA) (Fig. 4e). Here, *n* = 1 outliers were replaced across the vehicle, 0.01, and 0.1 mg/kg groups. There was a main effect of drug on latency to approach the chow bowl (*X*_2_ = 20.20, *p* = 0.0002, Friedman Test). Post hoc analysis showed 1 mg/kg increased latency to approach the bowl relative to vehicle (*p* = 0.0002) (Fig. 4f). Here, *n* = 1 outliers from the vehicle group, *n* = 2 from the 0.1 mg/kg group and *n* = 3 from the 0.3 mg/kg group were replaced.

**Fig. 5:**
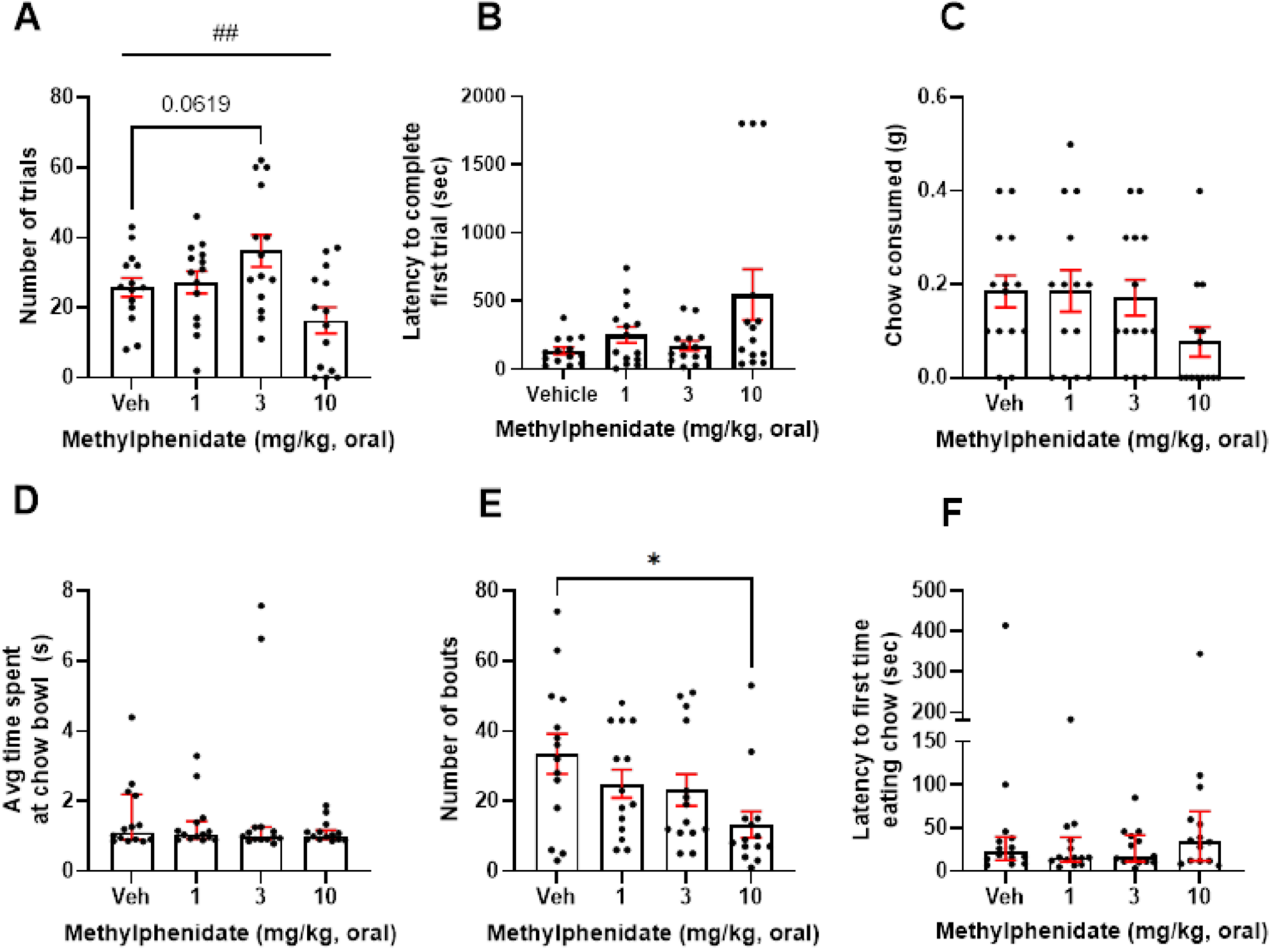
Methylphenidate increases high-effort option selection in a dose-dependent manner. Mice were dosed with 1–10 mg/kg methylphenidate and underwent the EfR task. **A** There was a main effect of methylphenidate on number of high-effort, high-value trials performed (*p* < 0.01) and trend towards 3 mg/kg increasing number of trials. **B** There was no effect of methylphenidate on latency to complete first trial, **C** chow consumed, **D** or average time spent at the chow bowl. **E** 10 mg/kg methylphenidate reduced number of chow bouts. **F** It had no effect on average time spent at the chow bowl or **G** latency to first approach bowl (*p* > 0.05). *p < 0.05, ^##^*p* < 0.01 (drug main effect). Bars are mean ± SEM or median ± interquartile range (**F**)

There was a main effect of methylphenidate (1–10 mg/kg) on number of trials (*F*_(2.067, 26.87)_ = 6.501, *p* = 0.0046, RM one-way ANOVA). Post hoc analysis revealed that 3 mg/kg trended towards increasing number of trials (*p* = 0.0619) (Fig. 5A). *N* = 1 outlier was replaced in the vehicle group. There was no effect on latency to complete first trial, though there was a trend towards an increase (*F*_(1.19, 15.46)_, *p* = 0.0621) (Fig. 5B). There was a trend towards a main effect of drug on chow consumed (*F*_(2.343, 30.46)_ = 2.611, *p* = 0.082, RM one-way ANOVA), and a main effect of drug on number of bouts at the chow bowl (*F*_(2.132,27.72)_ = 6.384, *p* = 0.0033, RM one-way ANOVA). Post hoc analysis showed that 10 mg/kg methylphenidate reduced the number of bouts to the chow bowl relative to vehicle (*p* = 0.0134) (Fig. 5C, E). Here, *n* = 1 outlier from the 10 mg/kg group was replaced. However, there was no effect of drug on average time at the chow bowl or latency to the chow bowl (*p* > 0.05) (Fig. 5D, F).

**Fig. 6:**
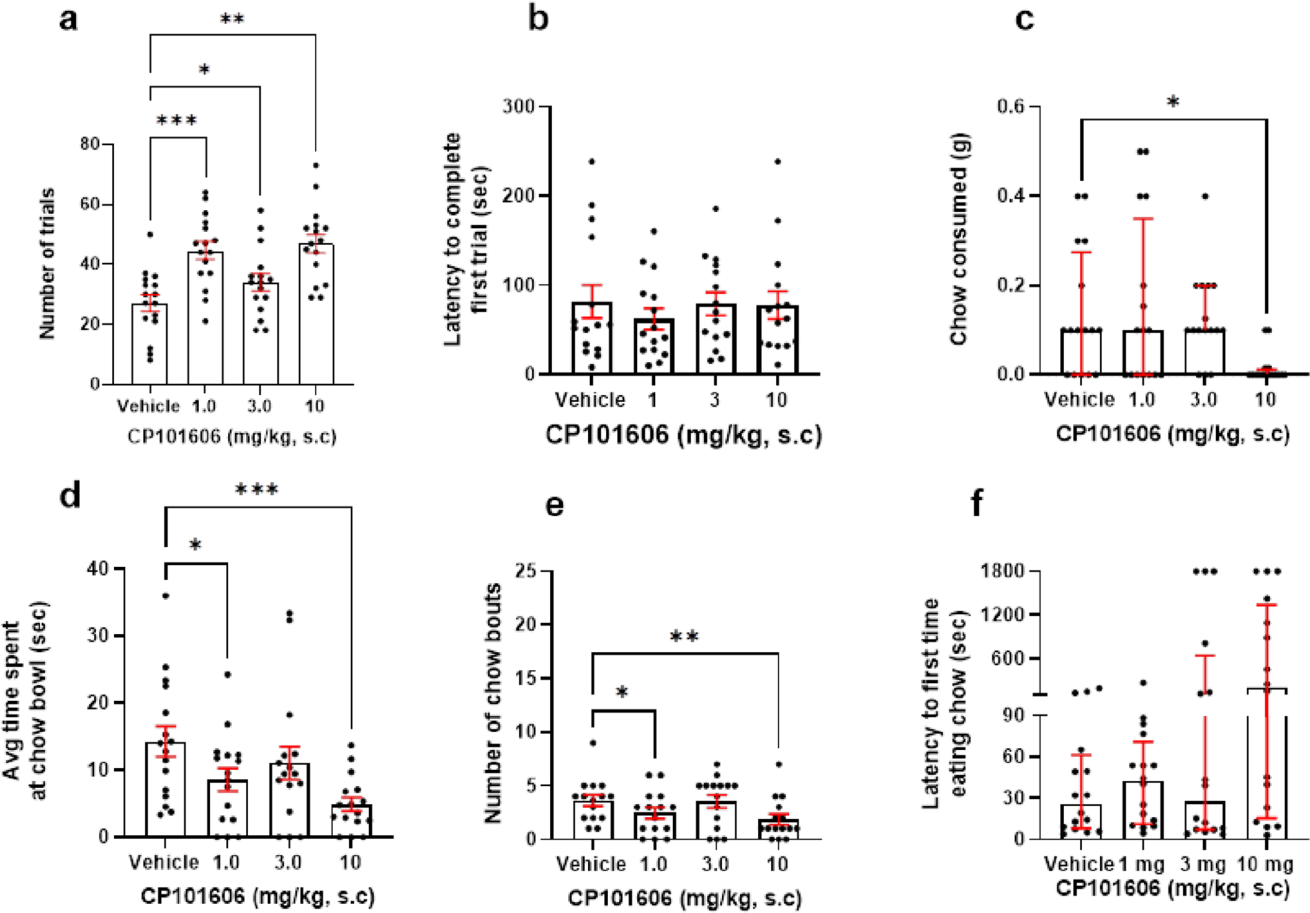
CP101-606 increases high-effort option selection in a dose-dependent manner. Mice were dosed with 1–10 mg/kg CP101-606 and underwent the EfR task. **a** 1, 3, and 10 mg/kg CP101-606 increased number of high-effort, high-value trials performed (*p* < 0.01, *p* < 0.05, and *p* < 0.001). **b** CP101-606 did not affect latency to complete first trial, **c** 10 mg/kg reduced chow consumed (*p* < 0.05). **d** 1 and 10 mg/kg decreased average time spent at the chow bowl (*p* < 0.05, *p* <0.001), as well as chow bouts (*p* < 0.05, *p* < 0.01). **f** It had no effect on latency to approach the bowl. *p < 0.05, **p < 0.01, ***p < 0.001. Bars are mean ± SEM or median ± interquartile range (**c** + **f**)

There was a main effect of CP101-606 (1–10 mg/kg) on number of trials completed (*F*_(1.647, 24.7)_ = 14.95, *p* = 0.0001, RM one-way ANOVA). Post hoc analysis revealed that 1, 3, and 10 mg/kg increased number of trials relative to vehicle (*p* = 0.0005, *p* = 0.0146, and *p* = 0.0019, respectively) (Fig. 6a). Here, *n* = 1 outliers from the 1 and 10 mg/kg groups were replaced. However, there was no effect of drug on latency to complete first trial (*p* > 0.05) (Fig. 6b). There was a main effect of CP101606 on amount of amount of chow consumed (*X*_2_ = 15.18, *p* = 0.0017, Friedman Test). Post hoc analysis revealed 10 mg/kg reduced amount of chow consumed relative to vehicle (*p* = 0.015) (Fig. 6c). Here, *n* = 1 outlier from the 1 and 3 mg/kg groups and *n* = 2 from the 10 mg/kg group were replaced. There was also a main effect of drug on average time spent at the chow bowl (*F*_(2.479, 37.18)_ = 6.933, *p* = 0.0015, RM one-way ANOVA). Post hoc analysis revealed that 1 and 10 mg/kg decreased duration at the chow bowl relative to vehicle (*p* = 0.0239 and *p* = 0.0002, respectively) (Fig. 6d). Here, *n* = 1 outlier was excluded across all groups. There was a main effect of drug on number of bouts at the chow bowl (*F*_(2.514, 35.19)_ = 5.134, *p* = 0.007, RM one-way ANOVA). Post hoc analysis showed that 1 mg/kg and 10 mg/kg reduced number of bouts at the chow bowl relative to vehicle (*p* = 0.0307, *p* = 0.006) (Fig. 6e). Here, *n* = 1 outliers across vehicle, 1, and 3 mg/kg were replaced and *n* = 2 at the highest dose. Finally, there was no effect of drug on latency to approach the chow bowl (Fig. 6f).

### Chronic corticosterone treatment does not affect effort-related behaviour

**Fig. 7:**
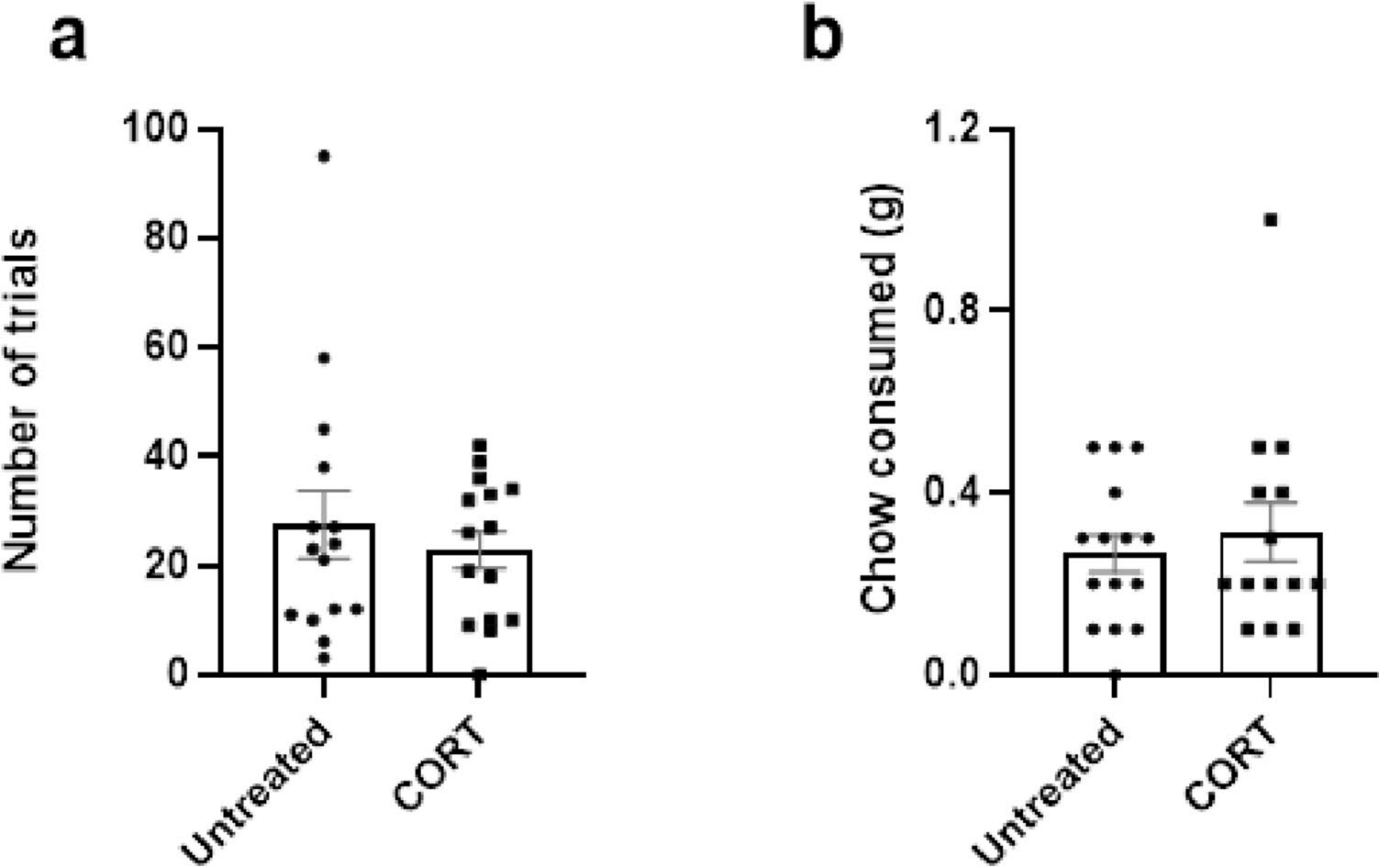
Chronic corticosterone has no effect on EfR performance. Mice were dosed with corticosterone for a period of 2 weeks and then underwent the EfR task. It had no effect on **a** number of trials or **b** chow consumed. Bars are mean ± SEM

There was no effect of a 2-week chronic CORT treatment on either number of trials or amount of chow consumed (*p* > 0.05, paired *t*-test) (Fig. 7a, b). *N* = 2 animals were excluded from chow consumed analysis due to outlying data. *N* = 1 animal was excluded from number of trials analysis due to outlying data.

## Discussion

Systemic administration of amphetamine, methylphenidate, and CP101606 in mice increased high-effort responses while administration of haloperidol decreased high-effort responses. Surprisingly, tetrabenazine did not appear to effect effort-based decision-making in this task in mice. While the drugs tested did not affect absolute consumption of the low-effort, low-value reward, video scoring revealed changes in chow bowl interaction for some of the treatments suggesting it can be used as a sensitive measure of low-effort, low-value reward engagement. Chronic low-dose corticosterone treatment did not impair effort-based decision-making in the EfR task. The following discussion considers how these results compare with data obtained from rats and contribute to our understanding of this task as a translational measure of motivational behaviour and effort-related decision-making.

### Mouse performance in the effort for reward task is sensitive to acute dopaminergic modulation

Consistent with previous studies using male and female rats (Salamone et al. 1991; Salamone et al. 1994; Griesius et al. 2020), we find that D2 antagonism following acute administration of 0.1 mg/kg haloperidol reduced the number of high-effort, high-value reward responses in male mice. This suggests that mouse performance in the task is similarly sensitive to reduction in DA signaling. Haloperidol did not suppress consumption of chow, suggesting that this effect is not driven by a general depletion in appetite. Video analysis found that haloperidol increased latency to engage with the bowl but did not affect latency to complete the first high-effort trial. This suggests that haloperidol may have reduced general exploration, which has been demonstrated in other studies using similar doses (Wiley 2008). EfR sensitivity to a reduction in dopaminergic transmission in rats has also been demonstrated following VMAT-2 inhibition using tetrabenazine, a treatment used in Huntington’s disease, and shown to induce depressive-like symptoms (Frank 2009). It has also previously been used in rodent studies to induce a depression-like phenotype and reliably reduced the number of high-effort responses made by rats at similar doses to the present study (Nunes et al. 2013; Griesius et al. 2020; Randall et al. 2014). Interestingly, our study found no effect of TBZ on effort-related performance in any of our output measures. There are a limited number of studies assessing the effect of TBZ in effort-related decision-making in mice. A study using a touchscreen-based effort-related decision-making task in mice found that TBZ reduced selection of the high-effort option, but at higher doses than tested here (6.0–8.0 mg/kg) (Yang et al. 2020b). Our lack of response may alternatively be driven by our experimental design. TBZ was administered at the end of the series of acute pharmacology experiments, and there is some evidence to suggest that repeated use of operant-based tasks may lead to procedural behaviour and a loss of task sensitivity. Consistent with this, a higher mean average of trials completed in the TBZ study was observed. This is unlikely to be driven by age, where a reduction in task performance has been observed (Jackson et al. 2021) and therefore may be a product of loss of sensitivity to effort requirement. Use of higher doses of TBZ and testing at the start of the battery may elucidate if the lack of effect is linked to these experimental factors or there are species differences which need to be taken into account.

EfR task performance has been shown to be sensitive to drugs which increase DA levels through blocking re-uptake and/or enhancing release. The psychostimulant amphetamine has been consistently shown to increase willingness to exert higher levels of effort for reward in humans (Soder et al. 2021; Wardle et al. 2011). In rats, low doses of amphetamine (0.25 mg/kg) increased high-effort responding in an effort discounting task (Floresco et al. 2008); however, had no effect on high-effort responses in the EfR task in female rats (Griesius et al. 2020). Utilising a dose response design in mice, we find the middle dose of amphetamine (0.3 mg/kg) selectively increased the number of high-effort responses, but this was not seen with the higher doses. This ‘inverted-U shape’ dose response curve is often seen with amphetamine and has been linked to doses which achieve optimal DA levels (O'Dell et al. 1999). At the highest dose tested, amphetamine did not increase responses relative to control and the latency to approach the chow bowl and receive the first pellet increased. This general reduction in both high- and low-reward engagement may be driven by the development of behaviours such as stereotypy observed at higher doses which may impact on task engagement.

We observe a similar pattern of effects using methylphenidate (MPH) which works via a similar mechanism to amphetamine but is only a re-uptake inhibitor and not a releasing agent. To our knowledge, MPH has not yet been tested in a preclinical EBDM paradigm. Here, we found a main effect of treatment on willingness to select the high-effort option although post hoc pairwise comparisons only found a trend level effect for the optimal dose. A reduced number of chow bouts at the higher dose was indicative of more generalised impairments, and there was no difference from saline for the high-effort, high-reward option at the highest dose. MPH is typically used in the treatment of ADHD, where it has been shown to increase willingness to exert effort for reward in patients (Addicott et al. 2019). However, it is also emerging as a promising treatment for apathy syndrome in neurodegenerative disease. A recent randomised control trial (RCT) found that treatment with MPH improved apathy symptoms in patients with Alzheimer’s disease (Mintzer et al. 2021).

Consistent with findings in rats, these data suggest that the effort for reward task is sensitive to dose-specific changes in DA modulation in mice. As such, we provide initial pharmacological validation of the EfR task as a measure of motivational state in mice. The effects of both amphetamine and methylphenidate and haloperidol reflect the effects observed in human subjects (Addicott et al. 2019; Soder et al. 2021; Frank 2009) and are consistent with predictive validity. It should be noted that amphetamine and methylphenidate do not have an isolated effect on the DA system. Both drugs additionally result in an increase in noradrenaline (NA) (Faraone 2018), and further studies using specific noradrenaline and dopaminergic reuptake inhibitors are needed to elucidate precise roles for these neurotransmitters in motivated behaviour.

### Effortful behaviour is potentiated by an NR2B selective NMDA antagonist

CP101-606 is a NR2B selective antagonist of the NMDA receptor which was investigated as a novel rapid-acting antidepressant and has been shown to have promising effects on motivation (Preskorn et al. 2008). Currently, there is no literature available on the effects of CP101606 in effort-related behaviours; however, it has been previously shown that 2.5–10 mg/kg CP101606 increased operant responding in a variable interval (VI) task in rats, suggesting that it may increase response motivation (Gilmour et al. 2009). Here, we show that CP101606 potentiates high-effort selection and reduces consumption of the low-effort chow at the higher dose. Video analysis revealed that the 1 and 10 mg/kg doses increased high-effort responses also reduced number of chow bouts and duration of time spent at the bowl. There was no effect on latency to complete first high-effort trial, suggesting a disengagement from the low-effort, low-value reward option specifically. This reduction in chow bowl engagement together with increase in high-effort selection together suggests a switch in choice behaviour. The mechanism for this potentiation in motivational state is unclear, but there is evidence for a close interaction between glutamatergic and dopaminergic signaling within the mesolimbic system (Tecuapetla et al. 2010).

Interestingly, other NMDA antagonists such ketamine have not shown positive motivational effects reducing the number of responses in a lever press VI task (Gilmour et al. 2009) and did not affect high-effort selection in the EfR task (Griesius et al. 2020). This discrepancy may be driven by distinct receptor-binding profiles, with ketamine additionally binding alternative NR2 subunit-containing receptors, as well as opioid, monoaminergic, nicotinic, cholinergic, and muscarinic receptors. Although further mechanistic studies are needed to understand these effects, the findings with CP101606 demonstrate that the EfR task is sensitive to a drug which does not directly target the dopamine system.

### Effortful behaviour was not sensitive to a chronic CORT treatment

Psychiatric disorders are driven by a complex interaction of factors that cannot fully be recapitulated by acute pharmacological manipulations typically used in behavioural tasks. Administration of corticosterone over an extended period of time is a well-validated pharmacological model of reward impairment (Hales et al. 2023). It may reflect the hypercortisolemia and circadian disruption seen in patients with MDD (Ma et al. 2018) or mimic chronic stress (Yang et al. 2015). Chronic CORT administration has previously been shown to impair motivation in the progressive ratio task in mice (Dieterich et al. 2019). Here, we find that chronic administration of a low dose of corticosterone does not affect performance in the EfR task. A previous study found that chronic CORT at higher doses (~ 10 mg/kg) had a limited effect of high-effort response selection in an operant-based EBDM task in mice (Dieterich et al. 2020). There are far clearer effects using a T-maze version of the task, where CORT robustly reduced the number of high-effort, high-reward arm choices. This suggests that the operant EfR task may not be a sensitive measure of a CORT-induced motivational deficit, and potentially, non-operant, more ethologically relevant tasks could provide a more sensitive readout of these deficits in this context. These data raise important questions about the sensitivity of the EfR tasks as a measure of motivational deficit within a phenotypic disease model. Currently, other models, e.g. psychosocial stress, maternal separation (Becker et al. 2021), have not been tested in EfR tasks.

It should be acknowledged that this study utilised males only, and to achieve greater translatability, both sexes should ideally be included.

## Conclusion

Using acute pharmacological manipulation of the DA and glutamatergic system in addition to testing a phenotypic model of depression, we investigated the validity of the EfR task as a translational measure of motivational state in mice. We show that, in line with rat data, mouse performance in the task is sensitive to DA manipulation and, in the case of amphetamine, aligns favorably with clinical data. However, the lack of TBZ effect suggests that there may be species differences or the need to test higher doses or that repeated use of the task may impact task sensitivity which should be considered within future experimental design. For the first time, we demonstrate robust increases in high-effort choices following treatment with CP101,606 suggesting that the task is also sensitive to an NR2B selective NMDA antagonist and glutamatergic modulation. Utilising novel video analysis of the chow-related behaviours provided a sensitive measure of engagement with the low-effort, low-value reward option that was not picked up by using the typical measure of absolute consumption of chow and suggests that this method generates additional behavioural data without using additional animals. We also provide a novel, open-access tool to assist in collecting these data. Finally, we find that the EfR task is not sensitive to a chronic CORT treatment, raising questions about its sensitivity as a measure of motivational state in non-acute pharmacological models.

### Supplementary information

ESM 1 (DOCX 171 kb)

## Data Availability

All data used in this manuscript are available and can be accessed on the Robinson Laboratory Open Science Framework upon acceptance https://osf.io/angk3/.

## Abbreviations

ADHD: Attention-deficit/hyperactivity disorder
COMT: Catechol-o-methyltransferase
CORT: Corticosterone
DA: Dopamine
EBDM: Effort-based decision-making
EEfRT: Effort Expenditure for Reward task
EfR: Effort for reward
GABA: Gamma-aminobutyric acid
MDD: Major depressive disorder
RDoC: Research Domain Criteria
TBZ: Tetrabenazine
VMAT-2: Vesicular monoamine transporter 2
