# Supplementary material for: Pharmacological characterisation of the effort for reward task as a measure of motivation for reward in male mice
Source: Psychopharmacology (Berl). 2023 Jul 21;240(11):2271–84. doi: 10.1007/s00213-023-06420-9 (PMC10593616; doi:10.1007/s00213-023-06420-9)
Supplement: Supplementary file 1 — (DOCX 171 kb) [file 213_2023_6420_MOESM1_ESM.docx]

**S1-Training data**


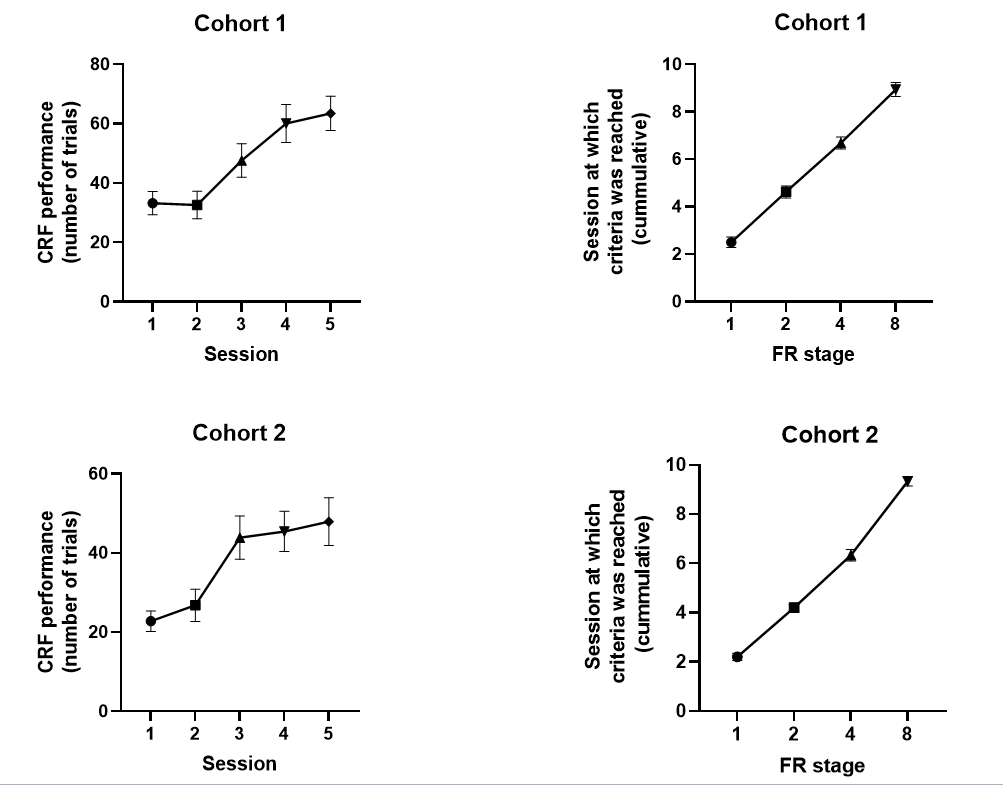


***S1- training data for both cohorts.*** *CRF- continuous reinforcement schedule. FR-fixed ratio.*


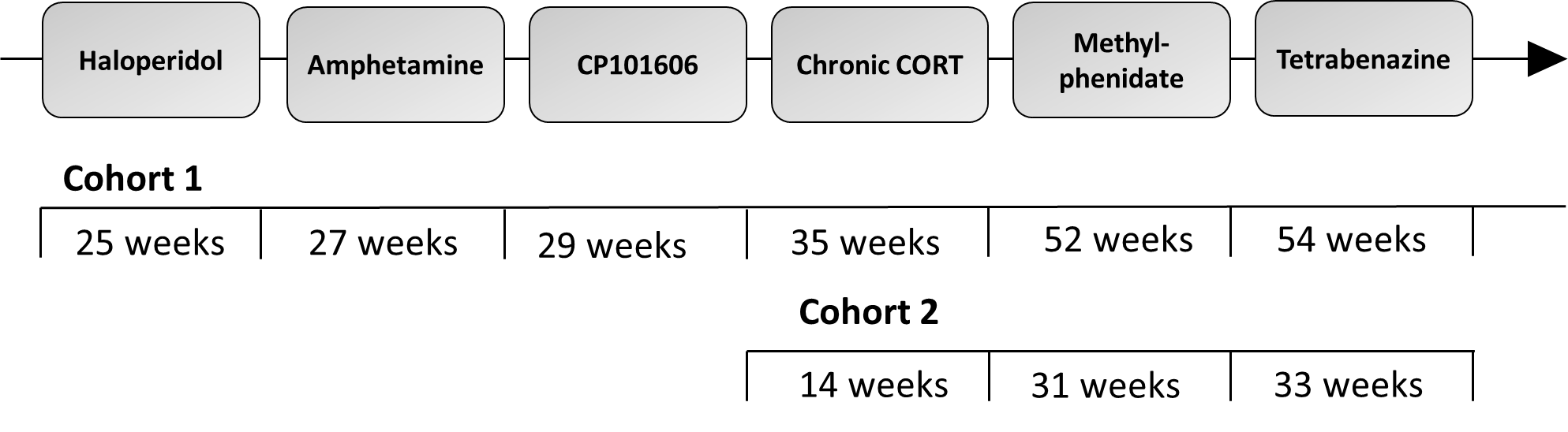
***S2***

***S2.*** *Testing order and mouse ages at the start of each drug study (cohorts 1 and 2).*


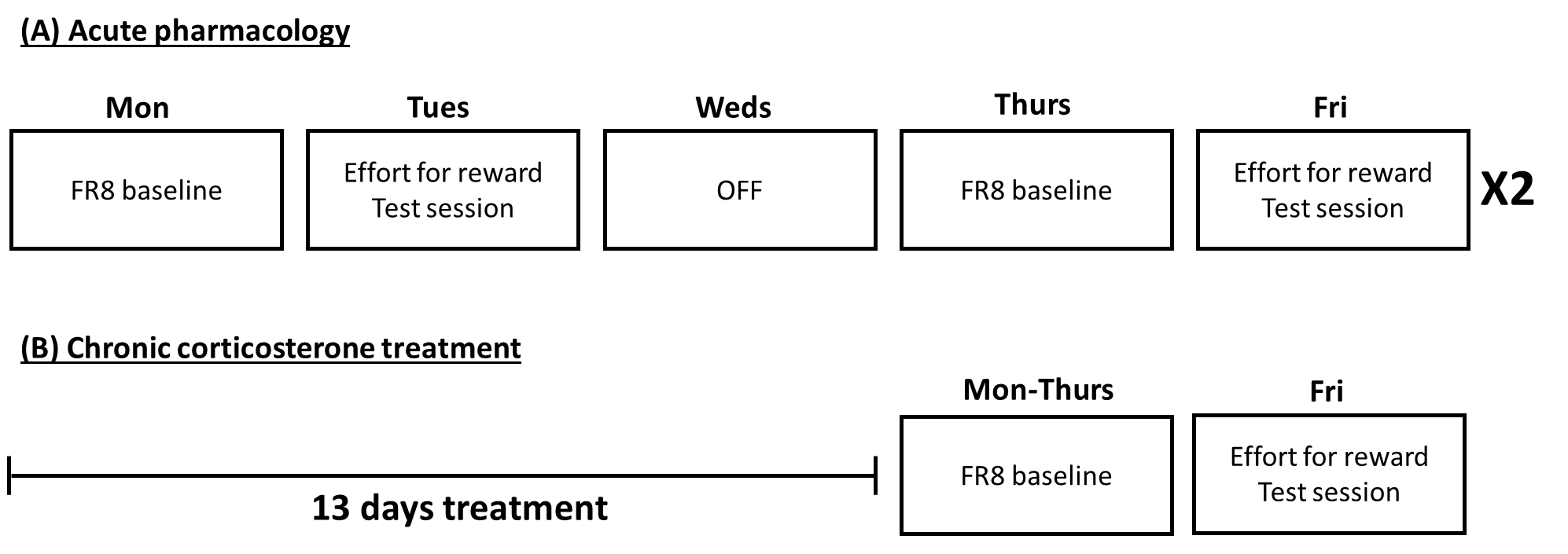
**S3**

***S3.*** *Structures of the acute and chronic pharmacology studies.* ***(A)*** *Each mouse received every dose of the drug in a within-subject, counterbalanced design spread across 4 test sessions (one dose per session). Each test session was preceded by an FR8 baseline day and a day off on the Wednesday.* ***(B)*** *Mice received 13 days of treatment before undergoing 4 days of re-baselining and a subsequent effort for reward test session.*
